# Genetic Variations in a PTEN/AKT/mTOR Axis and Prostate Cancer Risk in a Chinese Population

**DOI:** 10.1371/journal.pone.0040817

**Published:** 2012-07-18

**Authors:** Jiawei Chen, Pengfei Shao, Qiang Cao, Pu Li, Jie Li, Hongzhou Cai, Jian Zhu, Meilin Wang, Zhengdong Zhang, Chao Qin, Changjun Yin

**Affiliations:** 1 State Key Laboratory of Reproductive Medicine, Department of Urology, The First Affiliated Hospital of Nanjing Medical University, Nanjing, China; 2 Department of Molecular and Genetic Toxicology, Cancer Center of Nanjing Medical University, Nanjing, China; IPO, Inst Port Oncology, Portugal

## Abstract

**Backgroud:**

Genetic variations in a *PTEN*/*AKT*/*mTOR* signaling axis may influence cellular functions including cell growth, proliferation and apoptosis, and then increase the individual’s risk of cancer. Accordingly, we explore the association between single nucleotide polymorphisms (SNPs) of these genes and prostate cancer (PCa) in our Chinese population.

**Methods:**

Subjects were recruited from 666 PCa patients and 708 cancer-free controls, and eight SNPs in the *PTEN*/*AKT*/*mTOR* axis were determined by the TaqMan assay. Odds ratios (OR) and 95% confidence intervals (95% CI) were evaluated by logistic regression.

**Results:**

We observed significant associations between PCa risk and *mTOR* rs2295080 [*P* = 0.027, OR = 0.85, 95%CI = 0.74–0.98], and *AKT2* rs7254617 (*P* = 0.003, OR = 1.35, 95%CI = 1.11–1.64). When estimated these two SNPs together, the combined genotypes with 2–4 risk alleles (rs2295080 T and rs7254617 A alleles) were associated with an increased risk of PCa compared with 0–1 risk alleles, which was more pronounced among subgroups of age >71 years, smokers, drinkers and no family history of cancer. Results of stratified analyses by cliniopathological parameters revealed that the frequencies of the combined genotypes with 2–4 risk alleles in advanced stage were significantly higher than in localized stage(*P* = 0.022), but there was no significant association in Gleason score and PSA level.

**Conclusion:**

Our results indicate, for the first time that the two variants in *AKT2* and *mTOR*, particularly the joint genotypes with 2–4 risk alleles may influence PCa susceptibility and progression in Chinese, and the association appeared to be more strong in the subgroup of smokers and drinkers.

## Introduction

Prostate cancer(PCa) accounts for one-fourth of all tumors diagnosed in men in the United States, with an estimated 217,730 new cases and 32,050 deaths in 2010 [1]. In contrast, PCa morbidity is strikingly low in Asians. However, the occurrence of PCa has steadily increased in recent years in China [2]. Major risk factors for PCa are age, ethnic origin, lifestyle, environmental factors and genetic variants [3]. Although many people are exposed to these risk factors, only a small portion of the exposed individuals develop PCa, indicating that genetic variation partly contribute to the development and progression of PCa [4]. Hence, it is of great clinical significance to identify more molecular markers for detection and diagnosis of PCa. Among these molecular markers, the associations between gene polymorphisms and predisposition to PCa have been extensively investigated in recent years [5], [6], [7].

Dysregulation in cell proliferation, survival and growth drive the progressive transformation of normal cells toward a malignant phenotype. The *PTEN*/*AKT*/*mTOR* axis plays a crucial role in regulating cell growth, proliferation, apoptosis and drug responses [8], [9], [10]. Upstream, phosphatidylinositol 3′-kinase (*PI3K*) is deregulated through diverse mechanisms, including activation or overexpression of growth factors and hormones that bind receptor tyrosine kinases such as insulin-like growth factor receptor (*IGFR*), human epidermal growth factor 2 (*HER-2*), vascular endothelial growth factor (*VEGF*), and vascular endothelial growth factor (*PDGFR*) or mutation in *PI3K* [11], [12], [13], [14]. Then, the deregulated *PI3K* activate the kinase cascade of *AKT*-*mTOR*, in turn leads to phosphorylation of downstream elements including S6 kinase1(S6K1) and 4E-binding protein1(4E-BP1), generating the cell survival, growth, or apoptosis signal and resulting in protein translation by controlling ribosome biogenesis and ribosomal mRNA translation [15], [16]. The tumor suppressor *PTEN* is a plasma membrance associated lipid phosphatase acting as a negative regulator of *PI3K* signal by dephosphorylating phosphatidylinositol trisphosphate(*PIP3*) [17].

Previously, constitutive activation of the *PTEN/AKT/mTOR* signaling axis has been firmly established as a major determinant of tumor cell growth and survival in a multitude of solid tumors [18]. Similarly, substantial evidence have demonstrated that the *AKT/mTOR* was frequently over-expression and *PTEN* expression was markedly decreased in conditions of proliferative dysregulation and a multitude of solid tumors including PCa [19], [20]. Furthermore, uncontrolled *PTEN/AKT/mTOR* signaling also leads to poor clinical prognosis of lung, cervical, ovarian, esophageal and bladder cancer [8], [21], [22], [23], [24]. Due to the vital role of *PTEN/AKT/mTOR* axis in tumor pathophysiology, emerging research on the axis concern its potential as a target of rationally molecular anticancer therapies [15], [25].

As mentioned above, in spite of the well-known role of this pathway in tumor pathogenesis and progression, genetic variants in *PTEN/AKT/mTOR* genes had not been well studied. Overall, only some association studies assessed the relevance between polymorphism in the pathway and occurrence and prognosis of diverse type tumors, including bladder cancer, colorectal cancer and endometrial cancer [26], [27], [28], [29], [30]. For PCa, just one study had investigated tagging SNP in *mTOR* pathway and PCa risk in the European Prospective Investigation [31]. In light of the limited valuation of *PTEN/AKT/mTOR* gene variants, we sought to systemically investigate associations between single nucleotide polymorphisms (SNP), which may modify their expression or activity, and PCa susceptibility and progression in our case-control study in a Chinese population.

## Materials and Methods

### Ethics Statement

The study was approved by the Institutional Review Board of the Nanjing Medical University, Nanjing, China. At recruitment, written informed consent was obtained from all participants involved in this study.

### Study Population

From September 2003 to January 2010, a total of 666 patients with histopathologically confirmed and untreated PCa were prospectively recruited from The First Hospital of Nanjing Medical University, Nanjing, China. All these subjects were genetically unrelated ethnic Han Chinese and were from the Province of Jiangsu. In the interim, a total of 708 cancer-free controls were randomly recruited from healthy subjects who were seeking health care in the outpatient departments at the hospital. The selection criteria for the controls included no individual history of cancer and frequency matching to the cases on age (mean ±5 years). Before recruitment, a standard questionnaire was administered through face-to-face interviews by trained interviewers to collect demographic data, clinical data and related factors, including age, race, tobacco use, alcohol use, and family history of cancer. The clinical stage is classified according to TNM classification system. Localized prostate cancer can be detectable clinically on examination, but has not spread outside the prostate (T_1–2_N_0_M_0_). Advanced cancer means the cancer has spread through the prostatic capsule (T_3–4_N_X_M_X_ or T_X_N_1_M_X_ or T_X_N_X_M_1_). The Gleason score was estimated by pathologists working at the hospital using the Gleason scoring system. Smoking status, alcohol use, family history of cancer and their subgroups were defined as described previously [5]. After interview, each subject donated 5 ml of blood after written informed consent. The response rate for both case and control subjects was >85%.

### SNP Selection

Due to the huge number of SNPs in the human genome, the efficient selection of the SNPs most likely to contribute to phenotypic effects was the first challenge. Therefore, a preferential strategy was created using public databases that provide diverse information on the potential phenotypic risks of SNPs. We selected four core functional genes (*PTEN/AKT1/AKT2/mTOR*) in the axis. SNPs in *PTEN*/*AKT*/*mTOR* were based on HapMap data (http://hapmap.ncbi.nlm.nih.gov/) and PubMed data(http://www.ncbi.nlm.nih.gov/projects/SNP/). The potentially functional polymorphisms were identified according to the following standard: (1) located in the 5′ flanking regions, 5′ untranslated region (UTR), 3′ UTR, and coding regions with amino acid changes; (2) minor allele frequency (MAF) >5% in Chinese population; (3) associated with PCa risk in previous studies. When some of the SNPs were in complete linkage disequilibrium (r^2^ = 1), only one SNP was selected for genotyping. According to the standard, we selected eight SNPs. Among of them, two SNPs(rs11202607 and rs701848 in 3′UTR) in *PTEN*, two SNPs(rs2494750 and rs2498786 in 5′flanking regions) in *AKT1*, two SNPs(rs7254617 in 5′flanking regions and rs33933140 in 3′UTR) in *AKT2* and two SNPs(rs2536 in 3′UTR and rs2295080 in 5′flanking regions) in *mTOR*.

### DNA Extraction and Polymorphism Genotyping

Genomic DNA was extracted by proteinase K digestion and phenol–chloroform extraction. The sequence of primer and probe are available as requested. The eight selected SNPs of the PTEN/AKT/mTOR axis genes were determined using the TaqMan technology (Applied Biosystems, Foster City, CA, USA) according to the manufacturer’s instructions. The ABI PRISM 7900HT Fast Real-Time PCR System was used for the genotyping assay. Sequence Detection Systems software (SDS 2.3, Applied Biosystems) was used to automatically collect and analyze the data and to generate the genotype calls. For quality control, the genotyping analysis was performed in a blind manner. A 10% masked, random sample of cases and controls was randomly selected for repeated genotyping and all results were 100% concordance.

### Statistical Analysis

A goodness-of-fit chi-square test was used to detect the genotype distribution deviations from HWE. Differences in the distributions of demographic characteristics, selected variables, and frequencies of genotypes between cases and controls were analyzed using the Student’s t-test (for continuous variables) or chi-square test (for categorical variables). Odds ratios (ORs) and their corresponding 95% confidence intervals (CIs) were calculated for each SNP in association with PCa using an unconditional logistic regression model, with and without adjustment for confounding where appropriate. Potential modification of the effect of polymorphism on the risk of PCa was evaluated for the possible confounding factors by addition of interaction terms in the logistic model and by respective analyses of stratification of individuals determined by these factors. All statistical tests were two sided and carried out using the SAS 9.1.3(SAS Institute, Cary, NC) and *P* value <0.05 was regarded as statistically significant.

## Results

### Characteristics of Study Population

The selected characteristics of 666 patients and 708 controls are listed in **Table 1**. Overall, the cases and controls appeared to be well matched regarding age (*P* = 0.723). The mean age of PCa patients was 71.4 years and that of controls was 71.3 years. Yet, a significantly higher proportion of the cases were of smoking, drinking and positive for family history compared with controls (*P* = 0.013, *P* = 0.023, *P*<0.001, respectively). Furthermore, when according to the clinicopathologic parameters, 391(58.7%) of the 666 patients were in localized stage, the rest were in advanced stage. Approximately 58.9% of the patients PSA value >20 ng/ml and 41.1% ≤20 ng/ml. In addition, the percent of Gleason score from <7,  = 7 and >7 was 33.6%, 33.3% and 33.1%, respectively.

**Table 1:** Demographic and clinical variables of Prostate cancer cases and controls.

Variables	Cases (n = 666)		Controls (n = 708)		*P* *
	n	%	n	%	
Age (years) (Mean ± SD)	71.4±8.0		71.3±7.4		0.723
≤71	310	46.5	357	50.4	0.151
>71	356	53.5	351	49.6	
Smoking status					0.013
Never	280	42.0	345	48.7	
Ever	386	58.0	363	51.3	
Pack-years of smoking					<0.001
0	280	42.0	345	48.7	
0–22.5	149	22.4	184	26.0	
>22.5	237	35.6	179	25.3	
Drinking status					0.023
Never	469	70.4	537	75.8	
Ever	197	29.6	171	24.2	
Family history of cancer					<0.001
No	537	80.6	653	92.2	
Yes	129	19.4	55	7.8	
Clinical stage†					
Localized	391	58.7			
Advanced	275	41.3			
Gleason score					
<7	224	33.6			
= 7	222	33.3			
>7	220	33.1			
PSA (ng/ml)					
≤20	274	41.1			
>20	392	58.9			

* T-test for age distributions between the cases and controls; two-sided χ^2^ test for others selected variables between the cases and controls.

† Clinical staging according to the international TNM system for PCa. Localized: T_1–2_N_0_M_0_; Advanced: T_3–4_N_x_M_x_ or T_x_N_1_M_x_ or T_x_N_x_M_1_.

### Distribution of the *PTEN/AKT/mTOR* Genotype between Cases and Controls

**Table 2** summarized the genotype and allele distributions of SNPs among the cases and controls. The observed genotype frequencies of these eight SNPs in the controls were all in agreement with HWE (*P*>0.05). As shown in **Table 2**, for *mTOR* rs2295080, the genotype and allele distributions were crucially different between PCa cases and controls in the dominant mode (OR = 0.77, 95%CI = 0.61–0.98, *P* = 0.021). Moreover, the individuals carrying G allele had a significantly decreased risk of PCa compared with those carrying T allele (OR = 0.86, 95%CI = 0.74–0.98, *P* = 0.027). For *AKT2* rs7254617, the frequencies of the GG, GA and AA genotype among the cases were different from those among controls (*P* = 0.01), these discrepancies principally derived from a higher frequency of the GA genotype among cases by comparison with controls (25.8% versus 19.5%). Based on logistic regression analysis with adjustment for confounding factors, we used the most common GG genotype as reference, individuals harboring GA/AA genotype had a vital increased susceptibility to PCa compared with those carrying GG genotype(OR = 1.46, 95%CI = 1.13–1.88). However, no differences in the frequencies of the remaining SNPs in the *PTEN/AKT/mTOR* were observed between PCa cases and controls.

**Table 2:** SNPs in the *PTEN/AKT/mTOR* axis associated with the prostate cancer risk.

Polymorphisms	MAF	Case(n = 666)		Controls(n = 708)		*P* *	Adjusted OR^↑^	95%CI
		n	%	n	%			
PTEN rs11202607	0.096							
CC		532	79.9	577	81.5	0.328	1.00	reference
CT		124	18.6	126	17.8		1.07	(0.81–1.42)
TT		10	1.5	5	0.7		1.94	(0.64–5.84)
PTEN rs701848	0.419							
TT		212	31.8	235	33.2	0.655	1.00	reference
TC		329	49.4	353	49.9		1.06	(0.83–1.35)
CC		125	18.8	120	16.9		1.15	(0.84–1.58)
AKT1 rs2494750	0.321							
CC		317	47.6	331	46.8	0.730	1.00	reference
CG		269	40.4	299	42.2		0.94	(0.75–1.18)
GG		80	12.0	78	11.0		1.07	(0.75–1.54)
AKT1 rs2498786	0.182							
CC		429	64.4	480	67.8	0.342	1.00	Reference
CG		201	30.2	198	28.0		1.09	(0.93–1.03)
GG		36	5.4	30	4.2		1.32	(0.82–2.10)
AKT2 rs7254617	0.112							
GG		480	72.1	560	79.1	**0.0010**	1.00	reference
GA		172	25.8	138	19.5	**0.004**	1.44	(1.11–1.87)
AA		14	2.1	10	1.4	0.237	1.62	(0.70–3.74)
AA/GA		186	27.9	148	20.9	**0.002**	1.46	(1.13–1.88)
G allele		1132	85.0	1258	88.8	**0.003**	1.00	reference
A allele		200	15.0	158	11.2		1.35	(1.11–1.64)
AKT2 rs33933140	0.498							
AA		180	27.0	179	25.2	0.165	1.00	reference
AG		349	52.4	353	49.9		0.94	(0.72–1.22)
GG		137	20.6	176	24.9		0.76	(0.56–1.03)
MTOR rs2536	0.090							
TT		565	84.8	585	82.6	0.441	1.00	reference
TC		96	14.4	119	16.8		0.82	(0.61–1.11)
CC		5	0.8	4	0.6		1.26	(0.33–4.84)
MTOR rs2295080	0.234							
TT		429	64.4	413	58.3	0.068	1.00	reference
TG		209	31.4	259	36.6	**0.029**	0.77	(0.61–0.98)
GG		28	4.2	36	5.1	0.267	0.74	(0.44–1.24)
TG/GG		237	35.6	295	41.7	**0.021**	0.77	(0.62–0.96)
T allele		1067	80.1	1085	76.6	**0.027**	1.00	reference
G allele		265	19.9	331	23.4		0.85	(0.74–0.98)

Bold-faced values indicate significant difference.

* Two-sided χ^2^ test for either genotype distributions or allele frequencies between the cases and controls.

† Genotype-specific ORs were adjusted for age, smoking, drinking status and family history of cancer in logistic regression model; Allele-specific ORs were not adjusted; 95% CI: 95% confidence interval; MAF: minor allele frequency.

### Combined Analysis between *mTOR* rs2295080 and *AKT2* rs7254617 Polymorphisms and PCa Susceptibility

In light of possible combined effects from different variants or genotypes and potential interactions of *PTEN/AKT/mTOR* gene polymorphism on the risk of PCa, we then combined the two SNPs based on the number of the putative risk allele (rs2295080 T and rs7254617 G allele which appeared to be statistically associated with an increased risk of PCa). As listed in **Table 3**, we detected that the numbers of individuals with risk allele were different between cases and controls (*P* = 0.011). A larger percent of individuals carrying two or three risk alleles and fewer individuals with one risk alleles were discovered in the patients than in the controls. Furthermore, we dichotomized the combined risk alleles into two groups by the number of risk alleles and used the combined genotypes with 0–1 risk alleles as the reference, we found that genotypes with 2–4 risk alleles were in accordance with a statistically significantly increased susceptibility to PCa(OR = 1.41, 95%CI = 1.12–1.79, *P* = 0.004).

**Table 3:** Frequency distributions of the combined genotypes of rs2295080 T > G and rs7254617 G > A among the cases and controls, and the correlation to risk of PCa.

	Cases (n = 666)		Controls (n = 708)		*P* *	Adjusted OR (95% CI)†
	n	%	n	%		
Number of risk alleles‡						
0	21	3.1	31	4.4	**0.011**	1.00 (reference)
1	158	23.7	210	29.7	0.728	1.15 (0.63–2.10)
2	361	54.2	373	52.6	0.220	1.48 (0.82–2.65)
3	117	17.6	89	12.6	**0.034**	1.99 (1.05–3.77)
4	9	1.4	5	0.7	0.111	2.55 (0.69–9.39)
*P*_trend_					0.0004	
Recombined groups‡						
0–1	179	26.9	241	34.0	**0.004**	1.00 (reference)
2–4	487	73.1	467	66.0		1.41 (1.12–1.79)

Bold-faced values indicate significant difference.

* Two-sided χ^2^ test for the distributions between the cases and controls.

† Adjusted for age, smoking, drinking status and family history of cancer in logistic regression model; OR, odds ratio; 95% CI, 95% confidence interval.

‡ The 0–4 represents the numbers of risk alleles within the combined genotypes; the risk alleles used for the calculation were the rs2295080T and rs7254617A alleles.

### Stratification Analysis of the Association of Combined Genotypes and Risk of PCa

We further surveyed the effect of the combined genotypes of rs2295080 and rs7254617 polymorphisms on PCa risk stratified by age, smoking status, pack-years, drinking status and family history of cancer (**Table 4**). We found that the association between combined risk alleles and PCa risk were more evident in individuals who were more than 71 years(adjusted OR = 1.53, 95%CI = 1.10–2.13, *P* = 0.01), smoking(adjusted OR = 1.37, 95%CI = 1.01–1.88, *P* = 0.026), who were heavy smokers(pack-years>22.5, adjusted OR = 1.56, 95%CI = 1.03–2.37, *P* = 0.018), drinking(adjusted OR = 1.82, 95%CI = 1.16–2.85, *P* = 0.009) and without family history of cancer(adjusted OR = 1.44, 95%CI = 1.12–1.85, *P* = 0.006). However, no statistical evidence was found for any interactions between the combined genotypes and confounding factors (data not shown).

**Table 4:** Stratification analysis of the variant number of genotypes by selected variables in PCa patients and controls.

Variables	Cases(n = 666)		Controls(n = 708)		*P* *	Crude OR(95%CI)	Adjusted OR(95%CI)†
	Number of risk alleles‡		Number of risk alleles‡				
	0–1	2–4	0–1	2–4			
	n (%)	n (%)	n (%)	n (%)			
Total	179 (26.9)	487 (73.1)	241 (34.0)	467 (66.0)	**0.004**	1.40 (1.11–1.77)	1.41 (1.12–1.79)
Age (year)							
≤71	89 (28.7)	221 (71.3)	121 (33.9)	236 (66.1)	0.151	1.27 (0.92–1.77)	1.30 (0.93–1.83)
>71	90 (25.3)	266 (74.7)	120 (34.2)	231 (65.8)	**0.010**	1.54 (1.11–2.13)	1.53 (1.10–2.13)
Smoking Status							
Never	71 (25.4)	209 (74.6)	112 (32.5)	233 (67.5)	0.052	1.42 (1.00–2.01)	1.49 (1.04–2.13)
Ever	108 (28.0)	278 (72.0)	129 (35.5)	234 (64.5)	**0.026**	1.42 (1.04–1.93)	1.37 (1.01–1.88)
Pack-years							
0	71 (25.4)	209 (74.9)	112 (33.0)	233 (67.0)	0.052	1.42 (1.00–2.01)	1.49 (1.04–2.13)
0–22.5	45 (29.0)	110 (71.0)	57 (31.0)	127 (69.0)	0.697	1.03 (0.89–1.18)	1.02 (0.90–1.17)
>22.5	63 (29.1)	168 (70.9)	72 (40.2)	107 (59.8)	**0.018**	1.64 (1.09–2.47)	1.56 (1.03–2.37)
Drinking Status							
Never	130 (27.7)	339 (72.3)	177 (33.0)	360 (67.0)	0.072	1.28 (0.98–1.68)	1.28 (0.97–1.69)
Ever	49 (24.9)	148 (75.1)	64 (37.4)	107 (62.6)	**0.009**	1.81 (1.16–2.83)	1.82 (1.16–2.85)
Family history of cancer							
No	144 (26.8)	393 (73.2)	223 (34.2)	430 (65.8)	**0.006**	1.42 (1.10–1.82)	1.44 (1.12–1.85)
Yes	35 (27.1)	94 (72.9)	18 (32.7)	37 (67.3)	0.443	1.31 (0.66–2.59)	1.28 (0.98–1.68)

Bold-faced values indicate significant difference.

* Two-sided χ^2^ test for either genotype distributions or allele frequencies between the cases and controls.

† Adjusted for age, smoking status, drinking status and family history of cancer in logistic regression model; 95% CI: 95% confidence interval.

‡ The 0–4 represents the numbers of risk alleles within the combined genotypes; the risk alleles used for the calculation were the rs2295080T and rs7254617A alleles.

### Association between the Combined Genotypes and Progression of PCa

We performed stratification analysis to explore the association between polymorphisms of *PTEN/AKT/mTOR* genes and various clinicopathological characteristics of PCa. No significant differences were observed between SNPs in *PTEN/AKT/mTOR* gene and progression of PCa(data not shown). However, in the combined analysis (**Table 5**), there was an important correlation between the combined genotypes and clinical stage. We found that the frequencies of patients with 2–4 risk alleles in the advanced stage of PCa(77.8%) were much higher than in the localized stage(69.8%) (*P* = 0.022).

**Table 5:** Association between the combined genotypes of polymorphisms and clinicopathologic parameters of PCa.

Variable	Risk allele				*P* *	Adjusted OR (95% CI)*
	0–1		2–4			
	n	%	n	%		2–4 versus0–1
Clinical stage†					**0.022**	
Localized	118	30.2	273	69.8		1.0 (reference)
Advanced	61	22.2	214	77.8		**1.51 (1.05**–**2.16)**
Gleason score					0.328	
<7	67	29.9	157	70.1		1.0 (reference)
= 7	60	27.0	162	73.0		1.10 (0.72–1.67)
>7	52	23.6	168	76.4		1.37 (0.90–2.10)
PSA (ng/ml)					0.259	
≤20	80	29.2	194	70.8		1.0 (reference)
>20	99	25.3	293	74.7		1.22 (0.87–1.73)

Bold-faced values indicate significant difference.

* Adjusted for age, smoking status, drinking status and family history of cancer in logistic regression model; 95% CI: 95% confidence interval; OR: odds ratio.

† Clinical staging according to the international TNM system for PCa. Localized: T_1–2_N_0_M_0_; Advanced: T_3–4_N_x_M_x_ or T_x_N_1_M_x_ or T_x_N_x_M_1_.

## Discussion

Up to date, growing evidences have validated that the *PTEN/AKT/mTOR* axis are universally activated in numerous cancers including PCa, and inhibitors of these core genes are displaying great promise as the latent anticancer agents [32], [33], [34]. Meanwhile, the discovery of genetic variables in susceptibility to various cancers is presently topics focus of extensive epidemiological studies. Therefore, we estimate whether these SNPs may influence the susceptibility and progression of PCa.

In the current study with a relatively comprehensive selection of SNPs in the *PTEN/AKT/mTOR* axis, we surveyed the associations between the eight potentially functional SNPs and PCa risk. The major finding was that significant associations had been identified between SNPs in *AKT*2 gene rs7254617, *mTOR* gene 2295080 and PCa susceptibility. We observed that *AKT2* rs7254617A allele was associated with a significantly increased risk of PCa compared with G allele. For *mTOR* rs2295080 polymorphism, with the TT genotype as reference, the TG/GG genotypes were associated with statistically decreased risk of PCa. But we did not find any crucial associations between the remaining SNPs and PCa risk. Substantial evidences were proposed to support our results. Previously, mutations such as single amino acid changes which were clustered in the kinase active sites by genetic approaches using yeast conferred the constitutive hyperactivation of mTOR and somatic aberrations of *PTEN/AKT/mTOR* axis genes had been generally observed in various cancers including PCa [35], [36], [37], [38], [39]. Some immunohistochemistry studies had shown the elevated expressions of phospho-Akt, phospho-mTOR were observed in PCa tissues compared with benign prostatic hyperplasia(BPH) and high-grade prostatic intraepithelial neoplasia(HGPIN) tissues [40], [41], [42]. Moreover, a quantity of groups had being performing the preclinical trials to identify PCa that respond to the *PTEN/AKT/mTOR* inhibitors, either alone or in combination with other therapies in vitro and in vivo models [25], [43], [44], [45]. It was biologically plausible that genetic variations in the PTEN/AKT/mTOR axis may contribute to the PCa by influencing the expression of these core genes. Although we did not find any evidence published on the function of the two SNPs in the promoter of genes, given the location where these SNPs located and in silico analysis, the T to G base change of rs2295080 may change the predicted binding of the Cap and GATA-1 transcription factors, subsequently resulting in a decrease of the mTOR gene expression. Regarding rs7254617, we did not find the possible distinct binding with transcription factors by the web-based SNP analysis tool, TFSEARCH 1.3 (http://www.cbrc.jp/research/db/TFSEARCH.html). One possible explanation was that the association between rs7254617 SNP and PCa risk might be mediated by linkage disequilibrium with other causal loci which might affect the protein expression. Further functional experiments of these two SNPs were required to demonstrate our results. Additionally, despite multiple lines of evidence had verified that mutation of *PTEN* was indeed a commonly detected in different types of malignancy including PCa [46], [47], but our results indicated that there was no discrepancy in the genotype distributions of the two SNPs(rs11202607 and rs710848) in the functional regions of *PTEN* between PCa cases and controls, consistent with the results in PCa reported by Haiman *et al* [48], they lend conclusive support to our study showing no strong associations between common inherited variation in *PTEN* and PCa risk in multi-race including African-American, Native Hawaiian, Japanese, Latina and White man. One possibility was that the molecular mechanisms, such as epigenetic inactivation, loss of heterozygosity (LOH) and deletion of PTEN not SNP in *PTEN*, might lead to a true loss of function and subsequently contribute to PCa.

Furthermore, we observed that the joint genotypes with 2–4 risk alleles were significantly associated with the increased risk of PCa, supporting the perspective that the combined analysis could provide a comprehensive estimation of genetic susceptibility in candidate genes with low penetration and consequently improve risk prediction compared with a SNP. PCa was a complex disease attributed to multiple environmental and genetic factors, genetic variations in conjunction with environmental factors may provide better insight into the PCa carcinogenesis. Thus we made a subgroup analysis by the expose risk factors, and our results indicated that the joint effect of the two SNPs on risk of PCa were more evident in older than 71, which were supported by large body of evidence, which link DNA damage accumulation with age. We also found a higher risk in drinkers and smokers (particular in those pack-years more than 22.5), suggesting that the putative risk genotypes carriers were at greater risk if they had continuous exposure.

With respect to clinical prognosis, it is interesting to observe that the frequencies of the combined genotypes with 2–4 risk alleles in advanced stage were significantly higher than in localized stage, indicating that the putative risk genotypes were statistically associated with the progression of PCa. Large quantity of supposition had been proposed to provide biologic mechanisms by which the *PTEN/AKT/mTOR* axis could promote the evolution and metastasis of tumor. Wang *et al* reported that blocking the *CXCR6/AKT/mTOR* signaling pathway induces antimetastatic properties in PCa cells [49]. Shimizu *et al* demonstrated that the *AKT/mTOR/p70S6 kinase* pathways were involved in biological and clinical aggressiveness progression of PCa in PCa cells and surgical PCa specimens [50]. Sarkar *et al* found that expression level of p-Akt and p-mTOR were associated with the PCa development and poor progression by using immunohistochemistry, which is similar with the results reported by Malik *et al* [51]. Moreover, some studies validated that increased expression of p-AKT served as a predictor of biochemical recurrence after radical prostatectomy [52], [53]. Therefore, it is biologically plausible that the putative risk genotypes in *PTEN/AKT/mTOR* axis are implicated in the progression of PCa, indicating that this pathway is an exciting and rational molecular target for therapy in PCa. However, considering the limited small sample size, we can not rule out the possibility that these observed results are just due to chance. Our conclusion should be interpreted with caution.

In reviewing the results of this study, some limitations of this study should be noted. First, in view of our study is a retrospective hospital-based case-control design, the inherent selection bias and recall bias cannot be entirely excluded. However, the genotype frequency distributions of the selected SNPs among our controls were all confirmed to HWE, suggesting that the selection bias is unlikely to be substantial. Second, we can not obtain detailed survival data from all participants, which limit our ability to explore the relationship between the SNPs in PTEN/AKT/mTOR axis and prognosis and survival of PCa.

In conclusion, to our knowledge, the current study provides the evidence to systematically evaluate inherited genetic variation in the *PTEN/AKT/mTOR* axis in reference to the pathogenesis and progression of PCa. We have firstly certified that two SNPs in the *PTEN/AKT/mTOR* axis, particularly in combination, may confer the increased risk of PCa in the Chinese population, even after adjusting for the confounded risk factors. These conclusions may broaden our horizons in the biological basis of carcinogenesis of PCa. Nevertheless, our findings need to be confirmed by additional population-based prospective studies with detailed survival data in different ethnic groups.
